# Establishing a Sex-Specific Cut-Off for Temporal Bone Thickness in Transcranial Color-Coded Duplex Sonography

**DOI:** 10.3390/brainsci16030279

**Published:** 2026-02-28

**Authors:** Roberta Bitti, Barbara Schmid, Michael Lell, Konstantin Emil Thiel, Florian Steinmeyer, Milan Fritsche, Josefin Ammon, Panagiota Manava

**Affiliations:** 1 Department of Radiology and Nuclear Medicine, Nuremberg Hospital Campus North, Paracelsus Medical University (PMU), Prof.-Ernst-Nathan-Str. 1, 90419 Nuremberg, Germany; michael.lell@klinikum-nuernberg.de (M.L.); panagiota.manava@klinikum-nuernberg.de (P.M.); 2 Department of Neurology, Nuremberg Hospital Campus South, Paracelsus Medical University (PMU), Breslauer Str. 201, 90471 Nuremberg, Germany; barbara.schmid@klinikum-nuernberg.de; 3 Core Facility Biostatistics, Paracelsus Medical University (PMU), Strubergasse 21, 5020 Salzburg, Austria; konstantin.thiel@pmu.ac.at; 4 Technische Hochschule Nürnberg Georg Simon Ohm, Keßlerpl. 12, 90489 Nuremberg, Germany; florian.steinmeyer@th-nuernberg.de; 5 Applied Mathematics, Physics and Humanities, Nuremberg Institute of Technology, Keßlerpl. 12, 90489 Nuremberg, Germany; milan.fritsche@th-nuernberg.de; 6 Institute of Medical Physics, Nuremberg Hospital Campus North, Paracelsus Medical University (PMU), Prof.-Ernst-Nathan-Str. 1, 90419 Nuremberg, Germany; josefin.ammon@klinikum-nuernberg.de

**Keywords:** transcranial ultrasound, Bedside Imaging, computed tomography

## Abstract

**Highlights:**

**What are the main findings?**

- Temporal bone thickness measured on routine cranial computed tomography (CT) is the strongest predictor of temporal window failure in transcranial color-coded duplex sonography (TCCS).
- Sex-specific CT-based cut-off values (3.8 mm in men and 3.3 mm in women) reliably identify patients with a high probability of inadequate acoustic windows.

**What are the implications of the main findings?**

- CT-based pre-selection enables more efficient use of transcranial ultrasound by avoiding examinations with a high likelihood of failure.
- Implementation of sex-adapted thresholds may reduce operator dependency and improve workflow efficiency.

**Abstract:**

**Background/Objectives**: The aim of this study was to identify predictors of temporal window failure (TWF) in transcranial color-coded duplex sonography (TCCS) based on demographical and computed tomography (CT)-based parameters such as temporal bone thickness (TBT), and to define sex-specific thresholds for predicting TWF. **Methods**: We retrospectively analyzed a series of adult patients who underwent cranial CT and TCCS. Bitemporal TBT was measured in nine standardized regions on CT, and mean TBT per side was calculated. Temporal bone window (TBW) quality was graded with two semiquantitative TCCS scores assessing the visibility of the middle cerebral artery (MCA), contralateral temporal bone, mesencephalon, and ipsilateral sphenoid bone. Associations between TBT, sex, age, and TBW visibility were analyzed by correlation, chi-square tests, and logistic regression. **Results**: 200 patients (102 men, 98 women; mean age 68 ± 16 years) were enrolled. Mean TBT was 3.1 ± 0.7 mm (right) and 3.2 ± 0.7 mm (left). TBT correlated weakly with age (r = 0.15–0.18, *p* < 0.05) and was higher in women (*p* < 0.05). Age and sex influenced TBW visibility (*p* < 0.05) with small effect sizes. Increased TBT strongly predicted poor TBW (β ≈ –1.7, *p* < 0.001). Optimal TBT cut-offs predicting adequate TBW were 3.8 mm (men) and 3.3 mm (women), maximizing specificity (men: 0.95, women: 0.85) and negative predictive value (men: 0.87, women: 0.66). **Conclusions**: Advanced age and female sex were both associated with TWF. CT-assessed TBT represents a robust predictor of TCCS feasibility. Implementation of sex-specific TBT threshold values may facilitate patient pre-selection and improve procedural efficiency in neurosonographic diagnostics.

## 1. Introduction

Transcranial ultrasound, including techniques such as transcranial Doppler sonography (TCD) [1] and transcranial color-coded duplex sonography (TCCS) [2] is a non-invasive, cost-effective, and repeatable imaging modality that facilitates the evaluation of intracranial structures through the skull.

TCD [3] and TCCS [4] play a crucial role in the early diagnosis of vasospasm, particularly following aneurysmal subarachnoid hemorrhage (aSAH) [5,6], and are widely used for monitoring a variety of acute neurological conditions, including ischemic stroke [7], acute intracerebral hemorrhage [8], subdural hematoma [9], midline shift [10] and hydrocephalus [11]. Their bedside availability and real-time hemodynamic assessment make them especially valuable in acute neurocritical care settings [12]. TCCS is particularly useful in the evaluation of vascular pathologies, assisting in the diagnosis of intracranial aneurysms [13], arteriovenous malformations (AVM) [14], stenosis of the intracranial arteries [15], and Moyamoya disease [16]. It also has recognized roles beyond strictly vascular disorders, such as in the assessment of intracranial pressure elevation [17] and as a supportive tool in Parkinson’s disease evaluation [18]. Intrinsic advantages of TCCS, including the absence of ionizing radiation and the ease of brain insonation through the transfontanellar window, make it an especially valuable modality in pediatric imaging [19].

However, TCCS presents several practical limitations. Namely, it is highly dependent on both the operator’s skill and on the limited presence of viable acoustic windows [20]. This limitation appears to be inherent to transcranial ultrasound techniques in general, as comparable rates of temporal bone window (TBW) suitability have been reported for conventional TCD and imaging TCCS [21]. This suggests that, although TCD and TCCS differ in acquisition and display, temporal window adequacy remains a fundamental limiting factor. Variability in the thickness and density of the temporal bone can negatively impact image quality due to inconsistent signal attenuation and distortion [22]. Computed tomography (CT)-based studies have demonstrated that both the thickness and the structural homogeneity of the temporal bone are associated with acoustic window failure, indicating that cranial bone morphology is a measurable and clinically relevant determinant of transcranial ultrasound feasibility [23].

Current clinical protocols do not incorporate pre-selection of patients based on factors known to adversely affect the quality of TBW, despite longstanding evidence that characteristics such as advanced age and female sex are associated with reduced acoustic window adequacy [23,24]. Temporal window failure (TWF) is observed in approximately 30% of patients [25], rendering TCCS ineffective in these cases and contributing to diagnostic delays and suboptimal resource utilization. To address this issue, we conducted a cross-sectional study involving patients who underwent both TCCS and CT imaging. The objective was to identify demographic and anatomical predictors of TWF, using CT to assess the latter, with the goal of defining objective criteria to avoid unnecessary TCCS examinations in patients at high risk for inadequate acoustic windows.

## 2. Materials and Methods

### 2.1. Patient Recruitment

We retrospectively analyzed the data of adult patients who received a CT scan of the skull to rule out acute intracranial pathologies such as ischemic stroke or hemorrhage between September and November 2021 at the department of Radiology of the Klinikum Nuremberg (Nuremberg, Germany), a tertiary academic center, and assessed them for eligibility. All patients above 18 years of age who had an indication for TCCS after neurologic examination were included in the study. The examiner was blinded to CT results but was not blinded to the patients’ clinical status. This study was conducted under the principles of the Declaration of Helsinki. The need for informed consent was waived under the Bavarian Hospital Law (Bayerisches Krankenhausgesetz Art. 27 Abs. 4) and the General Data Protection Regulation (EU) 2016/679 (GDPR) due to the study’s retrospective and anonymous design.

### 2.2. Ultrasound Methods

TCCS was performed bilaterally by a single, highly experienced neurologist (B.S., over 20 years of experience) to ensure consistency and minimize variability associated with operator-dependent factors. TCCS were conducted as part of routine clinical care, primarily for the evaluation and exclusion of intracranial arterial vasospasm. TCCS was performed with an Affiniti 70 ultrasound device (Philips BV, Eindhoven, The Netherlands) with a 2 MHz sectorial probe with a scanning depth of 16 cm using the B-mode and Color Doppler modality. The image brightness, contrast and time gain compensation settings were adapted as needed for each patient. The transducer was placed temporally at the orbitomeatal line. First, the brain stem was visualized to obtain a landmark for orientation. The transducer was then angled approximately 10° upward to visualize the contralateral skull bone. The probe was tilted according to the patients’ anatomy to enhance visualization of intracranial structures across the mesencephalic, diencephalic, and ventricular planes. We established two scores to evaluate TBW, both based on visibility of various intracranial structures by TCCS (Table 1). The first score evaluated the visibility of the middle cerebral artery (MCA) on a discrete 0 to 3 scale (i.e., MCA score, Table 1). The second score, the TMS score, assessed the visibility of the contralateral temporal bone, the mesencephalon, and the ipsilateral sphenoid bone on a discrete 0 to 3 scale (i.e., TMS score, Table 1).

### 2.3. CT Methods

Cranial CT scans were performed as part of routine clinical care and per protocol. All examinations were acquired using the same CT scanner (Somatom Definition AS64, Siemens Healthineers, Erlangen, Germany) with identical scan parameters (120 kV, 200 mA, xCARE, helical mode, 512 × 512 matrix, reconstructed slice thickness of 1 and 4 mm in soft and hard kernel, source-to-detector distance 1085.6 mm, source-to-patient distance 595 mm). All measurements of the TBT were performed manually using the measurement tools of a Picture Archiving and Communication System (PACS) workstation (Visage Imaging Inc., San Diego, CA, USA). TBT was measured on high-resolution bone reconstructions (H70h kernel) with a slice thickness of 1 mm and an in-plane pixel spacing of 0.4 × 0.4 mm, reconstruction interval of 1 mm, resulting in an anisotropic voxel size of 0.4 × 0.4 × 1 mm. No intravenous contrast agent was administered.

**Figure 1:**
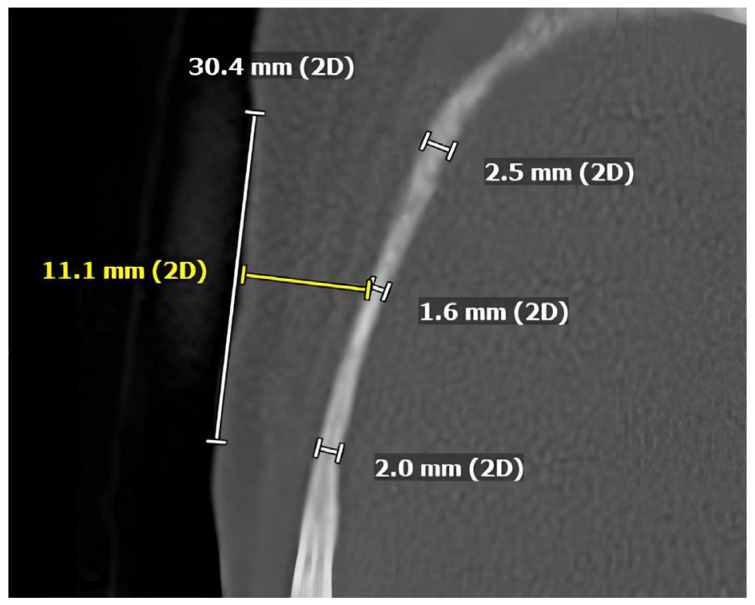
Axial CT-plane demonstrating how temporal bone thickness (TBT) measurements were performed. For illustrative purposes, this image shows three measurement points on a single axial slice corresponding to the most basal level (BF, BM, BO).

**Figure 2:**
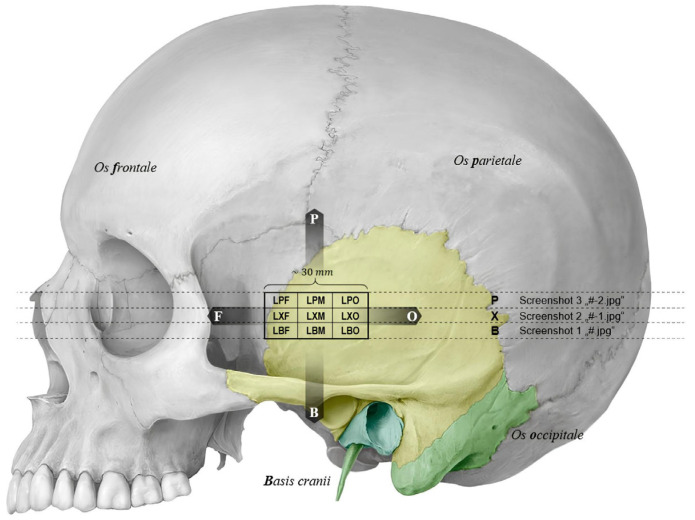
Schematic representation of the temporal bone where the probe was placed to perform transcranial color-coded duplex sonography (TCCS) and the 9 areas in which TBT was measured (mm). The image illustrates the 9 regions of the left temporal bone: left parietofrontal (LPF), left intermediate frontal, (LXF), left basofrontal (LBF), left central parietal (LPM), left central intermediate (LXM), left central basal (LBM), left parietooccipital (LPO), left inter-mediate occipital (LXO), left basooccipital (LBO).

Temporal bone thickness (TBT) was measured a in millimeters (mm) at the same sites where TCCS was performed (Figure 1). To account for spatial heterogeneity of TBT at the insonation site, the broader area on the temporal bone where the ultrasound transducer was placed during examination was subdivided into nine subregions per side: parietofrontal, intermediate frontal, basofrontal, central parietal, central intermediate, central basal, parietooccipital, intermediate occipital, basooccipital (Figure 2). TBT was measured separately in each subregion, and the mean of the nine measurements was used as the representative value per side. This approach was chosen to reduce local anatomical variability and improve measurement precision through spatial averaging.

### 2.4. Statistical Analysis

Descriptive data were analyzed using means and standard deviations for continuous variables, median and interquartile ranges for non-continuous variables, and percentages for categorical variables, as appropriate. Pearson’s correlation was calculated to assess the relationship between TBT and age. Welch’s two-sample t-tests were used to compare mean TBT values between sexes. Because each patient contributed bilateral measurements, left and right observations were not statistically independent; therefore, analyses were performed separately for each side and interpreted jointly.

Two groups were defined to divide measurements with good and insufficient TBW based on the TMS and MCA scores. When either TMS or MCA were <2, TBW was judged as “poor”, while when both scores were ≥2, TBW was judged as “good”. These two categories were assessed bilaterally. Chi-square tests were used to determine the relationship between visibility and sex, whereas logistic regression models were employed to examine the impact of age on visibility. Furthermore, to examine whether TBT independently predicts visibility, two additional logistic regression models were performed using TBT as the independent variable. The models evaluated the impact of right and left TBT on visibility outcomes. A threshold of α = 0.05 was used to determine statistical significance.

Finally, to identify the optimal TBT threshold for predicting good TBW, performance metrics including specificity, sensitivity, negative (NPV) and positive predictive value (PPV) for the outcome of “good visibility” were calculated for different TBT cut-off values in mm. One cut-off was selected for men and one for women, prioritizing high specificity and NPV while maintaining acceptable levels of sensitivity and PPV.

Receiver operating characteristic (ROC) curve analysis was performed to evaluate the diagnostic performance of the studied predictors and to determine optimal cut-off values.

R version 4.4.1. (R Core Team (2024). R: A Language and Environment for Statistical Computing. R Foundation for Statistical Computing, Vienna, Austria) statistical software was used for all data analyses.

## 3. Results

### 3.1. Patients Characteristics

- A total of 200 patients (102 men, 98 women) with a mean (±SD) age of 68.0 (16.2) years were enrolled and received bilateral TCCS and a brain CT scan, providing 200 cranial CT scans (one per patient) and 400 TCCS assessments (bilateral examinations, one per side in 200 patients)

### 3.2. TBT Distribution According to Age and Sex

**Figure 3:**
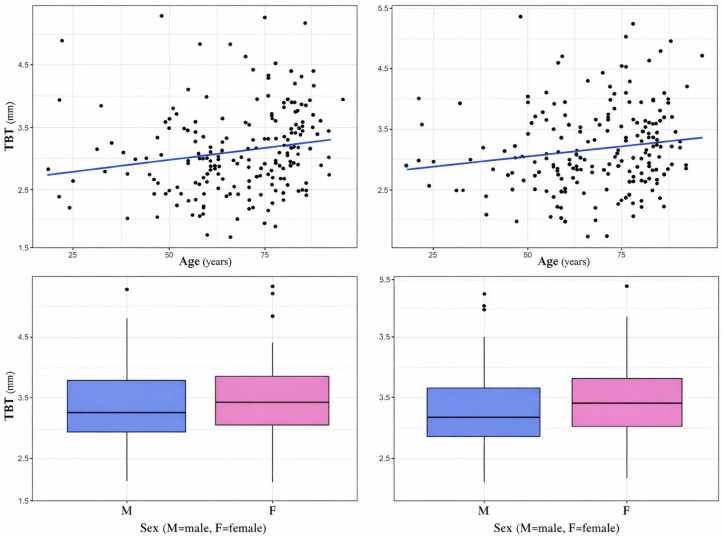
Upper panels: Scatter plot and correlation line illustrating the relationship between age and TBT. Each dot represents an individual measurement. Lower panels: Box plot comparing TBT on the both sides of the head between men (0) and women (1). Each box represents the interquartile range (IQR), with the horizontal line indicating the median. Whiskers represent values within 1.5× IQR from the quartiles, and individual dots denote outliers.

- The mean TBT on CT was 3.1 (0.7) mm and 3.2 (0.7) mm on the right and left side, respectively.
- There was a weak but statistically significant positive correlation between TBT and age. For TBT on the right side, the Pearson correlation coefficient was r = 0.18, *p* < 0.05 (Figure 3), for TBT on the left side, it was r = 0.15 (*p* < 0.05) (Figure 3).
- Women had higher TBT compared to men. For TBT on the right side, TBT was higher in women (mean TBT: 3.2 ± 0.7 mm) compared to men (mean TBT: 3.0 ± 0.7 mm) but differences were not statistically significant (*p* = 0.14) (Figure 3). Similarly, on the left side women had a mean TBT of 3.3 ± 0.7 mm and men of 3.0 ± 0.7 mm, but differences were in this case statistically significant (*p* < 0.05) (Figure 3).

### 3.3. MCA- and TMS Score Distribution

- The median (IQR) MCA score was 2 (0–3), while the median (IQR) TMS score was 2 (1–3). The distribution of TCCS by MCA and TMS scores is reported in Table 2. For the MCA score, class 3 (complete visibility) represented the most frequent category on the right side, whereas on the left side both class 0 and class 3 were commonly observed. Class 1 was less frequently represented.
- For the TMS score, class 3 was also the most represented category on the right side. On the left side, the distribution was more evenly spread across categories, with class 1 and class 3 occurring most frequently. Lower score categories (0–1) were observed on both sides for both scoring systems.

### 3.4. Impact of Age, Sex and TBT on Visibility

**Figure 4:**
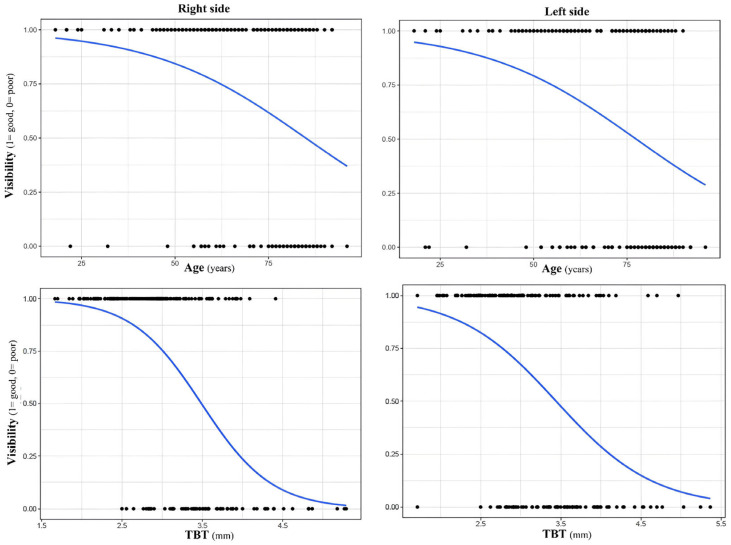
Logistic regression model showing the probability of good visibility as a function of age (in years) and TBT (in mm). Each dot represents an individual measurement (1 = good visibility, 0= poor visibility). The blue curve indicates the predicted probability of visibility.

- Age significantly influenced visibility on both sides of the head, but the size of the influence was modest. Logistic regression models revealed a small but significant correlation on both the right side (β = −0.05, standard error (SE) = 0.01, *p* < 0.05) (Figure 4) and on the left side (β = −0.05, SE = 0.01, *p* < 0.05) (Figure 4).

- A chi-square test revealed a significant impact of sex on visibility both on the right (X^2^ = 11.27, *p* < 0.05) and left side of the head (X^2^ = 11.58, *p* < 0.05).
- Univariate logistic regression analyses revealed that increased TBT was a very good predictor of poor TBW on both sides (right side: β = −1.78, SE = 0.30, *p* < 0.05; left side: β = −1.65, SE = 0.29, *p* < 0.05, Figure 4).

### 3.5. Sex-Specific TBT-Cutoffs to Predict Visibility

**Figure 5:**
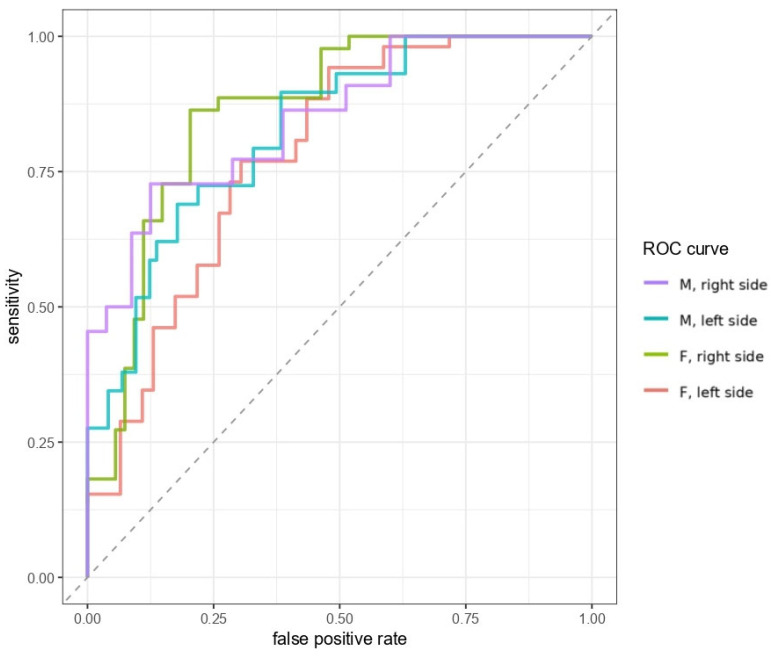
Receiver operating characteristic (ROC) curves illustrating the diagnostic performance of sex-specific TBT thresholds for predicting temporal bone window (TBW) visibility on TCCS, shown separately for men and women and for both sides.

- Performance metrics of different TBT cutoffs for the prediction of good TBW by sex and side are shown in detail in Table 3 and Table 4. Two sex-specific cutoffs were identified to optimize specificity and NPV. In men, a TBT cut-off of 3.8 mm was chosen, resulting in high specificity (right: 0.95, left: 0.93) and NPV (right: 0.87, left: 0.78), while retaining acceptable sensitivity (right: 0.5, left: 0.34) PPV (right: 0.73, left: 0.67). A cut-off of 3.3 mm was selected for women, with similar specificity (right: 0.85, left: 0.72), NPV (right: 0.74, left: 0.66), sensitivity (right: 0.66, left: 0.67) and PPV (right: 0.76, left: 0.73). ROC curves illustrating the diagnostic performance of sex-specific TBT thresholds are visualized in Figure 5.

## 4. Discussion

Since the implementation of TCCS in routine protocols for the evaluation of intracranial vascular pathology, interest has grown in delineating the patient characteristics that determine its applicability and diagnostic yield. In emergency settings, non-contrast cranial CT is routinely performed to exclude acute ischemic stroke or intracranial hemorrhage. This study aimed to establish a CT-derived cut-off value to predict the likelihood of successful downstream TCCS examination. In line with previous literature, the TBT of our cohort was higher with increasing age and in the female sex [23,25]. However, age and sex per se were poor predictors of visibility, with only a minor impact on MCA and TMS scores, indicating that demographic variables alone are insufficient to reliably predict acoustic window adequacy at the individual patient level. In contrast, TBT represents a direct structural measure of ultrasound attenuation and emerged as the major predictor of TWF, with logistic regression models indicating that variations in TBT explain a substantial proportion of the variability in TWF.

Based on this observation, we sought to define a CT-based threshold value above which TCCS should be avoided due to the high probability of TWF. By defining the TBT value based on the mean of 9 acquisition at different parts of the temporal bone, the accuracy of the measurement was increased compared to previous studies. Furthermore, two scores were used to define TWF not only in relationship to vascular but also to bone and cerebral structures.

Given that sex is a known independent predictor of poor acoustic window quality, we derived sex-specific cut-offs. A threshold of 3.8 mm for men and 3.3 mm for women yielded the highest specificity and NPV, while maintaining acceptable sensitivity and PPV. We prioritized specificity and NPV to minimize the exclusion of patients with adequate temporal windows, as TCCS is intended as a first-line diagnostic tool and unnecessary exclusion could cause misuse of CT or MRI. ROC analysis showed that sex-specific TBT thresholds provide good discrimination for temporal window adequacy, supporting their use as a rule-out tool.

Compared with previous CT-based studies, the present work provides clinically applicable, sex-specific threshold values derived from systematic spatial sampling of the temporal bone and validated against standardized visibility scores. Because cranial CT is routinely available in acute neurological care, this approach translates anatomical assessment into a practical decision-support framework that may help avoid non-diagnostic TCCS examinations while preserving access in patients with a high likelihood of adequate acoustic window.

There are some limitations to this study. First, there are technical constraints related to CT-based evaluation. TBT was measured manually in mm, which may introduce observer-related variability. The influence of temporal bone density and structural homogeneity was not assessed. However, the available evidence on this topic remains scarce and conflicting [22,24], with no clear indication of a significant impact on acoustic window visibility. Second, TCCS in our study was performed without contrast agent, which is known to improve visibility [26,27]. Nonetheless, this is not always accessible in acute setting and TCCS is mostly performed without contrast agent.

All examinations were conducted by a single experienced neurologist to minimize operator-dependent variability and ensure methodological consistency. However, this approach precluded assessment of inter-rater reliability, and the reproducibility of the findings across different examiners cannot be directly determined. Although no formal intra- or inter-observer reproducibility analysis was performed, the measurement protocol incorporated spatial sampling across nine predefined subregions, and averaging these values reduces random variability and local anatomical heterogeneity, thereby improving precision of the thickness estimate. Additionally, while the examiner was blinded to CT-derived measurements, the clinical status of the patients was known during TCCS examinations. This lack of full blinding may have introduced observational bias. This study used consecutive retrospective sampling, providing a real-world clinical cohort with a broad age distribution approximating the general adult population. As the aim was to evaluate the predictive value of CT-derived TBT for temporal window adequacy, the proposed thresholds should be interpreted as clinically derived decision-support values. Lastly, the study population consisted predominantly of Caucasian patients, which may limit the generalizability of the findings to other ethnic groups. Differences in cranial bone structure and reported rates of TWF across Asian and African populations [28] suggest that the proposed thresholds may not be directly transferable without further validation in more diverse cohorts. Future studies in ethnically diverse populations will be needed to confirm the broader applicability of the results.

## 5. Conclusions

In conclusion, to the best of our knowledge, we described for the first time sex-adapted thresholds for the early identification of patients with high risk of TWF based on the visibility of both vascular and non-vascular intracranial structures with TCCS. These findings could facilitate clinical decision-making and the establishment of more cost- and time-effective protocols to assess intracranial pathology using TCCS.

## Figures and Tables

**Table 1:** Classification of the temporal bone window according to the two scores based on the visibility the middle cerebral artery (MCA score) and on the visibility of different intracranial structures (temporal bone, mesencephalon, sphenoid bone, TMS score).

**MCA Score**	**Clinical Definition**
Class 0	No visibility of the MCA
Class 1	Poorly visible MCA, defined as segmentary view of <50% of the artery
Class 2	Good visibility of the MCA, defined as a visibility of more than 50% of the artery
Class 3	Complete visibility of the MCA
**TMS Score**	**Clinical Definition**
Class 0	Temporal bone (contralateral), the mesencephalon or sphenoid bone (ipsilateral) are not visible
Class 1	1 structure is visible
Class 2	2 structures are visible
Class 3	All three of structures are visible

MCA: middle cerebral artery score; TMS: temporal bone, mesencephalon, sphenoid bone score.

**Table 2:** Distribution of TCCS findings according to MCA and TMS scores (classes 0–3). Frequencies are reported separately for the right and left sides.

Grade	MCA-Score Right Side, n (%)	MCA-Score Left Side, n (%)	TMS-Score Right Side, n (%)	TMS-Score Left Side, n (%)
0	56 (28.0%)	70 (35.0%)	26 (13.0%)	27 (13.5%)
1	23 (11.5%)	16 (8.0%)	58 (29.0%)	65 (32.5%)
2	40 (20.0%)	52 (26.0%)	35 (17.5%)	41 (20.5%)
3	81 (40.5%)	62 (31.0%)	81 (40.5%)	67 (33.5%)

**Table 3:** Cut-off values in mm measured in men and their performance metrics in predicting the outcome of “good visibility”.

Cutoff	Specificity	Sensitivity	NPV	PPV
**Right**				
2.5	0.34	1.00	0.96	0.29
2.6	0.40	0.91	0.94	0.29
2.7	0.49	0.86	0.93	0.31
2.8	0.53	0.86	0.91	0.33
2.9	0.61	0.77	0.89	0.35
3	0.71	0.73	0.90	0.40
3.1	0.76	0.73	0.91	0.44
3.2	0.79	0.73	0.91	0.48
3.3	0.83	0.73	0.92	0.53
3.4	0.85	0.73	0.92	0.57
3.5	0.88	0.64	0.90	0.58
3.6	0.91	0.50	0.87	0.58
3.7	0.95	0.50	0.87	0.69
3.8 **	0.95	0.50	0.87	0.73
3.9	0.96	0.45	0.87	0.77
4	0.98	0.45	0.86	0.83
**Left**				
2.5	0.34	0.97	0.93	0.36
2.6	0.37	0.93	0.93	0.37
2.7	0.47	0.93	0.94	0.41
2.8	0.51	0.90	0.93	0.41
2.9	0.62	0.79	0.88	0.44
3	0.67	0.72	0.86	0.46
3.1	0.78	0.69	0.86	0.56
3.2	0.79	0.69	0.85	0.57
3.3	0.82	0.62	0.85	0.58
3.4	0.86	0.59	0.84	0.61
3.5	0.88	0.52	0.82	0.63
3.6	0.90	0.38	0.79	0.58
3.7	0.92	0.38	0.79	0.65
3.8 **	0.93	0.34	0.78	0.67
3.9	0.93	0.31	0.77	0.64
4	0.96	0.28	0.77	0.73

NPV: negative predicted value; PPV: positive predicted value. ** indicates the selected optimal cutoff value.

**Table 4:** Cutoff values in mm measured in women and their performance metrics in predicting the outcome of “good visibility”.

Cutoff	Specificity	Sensitivity	NPV	PPV
**Right**				
2.5	0.30	1.00	1.00	0.54
2.6	0.35	1.00	1.00	0.56
2.7	0.44	1.00	1.00	0.58
2.8	0.48	0.98	0.96	0.61
2.9	0.54	0.89	0.85	0.61
3	0.67	0.89	0.88	0.68
3.1	0.74	0.86	0.85	0.73
3.2	0.80	0.73	0.78	0.74
3.3 **	0.85	0.66	0.74	0.76
3.4	0.85	0.55	0.70	0.75
3.5	0.89	0.48	0.67	0.78
3.6	0.91	0.39	0.64	0.77
3.7	0.91	0.34	0.63	0.75
3.8	0.93	0.27	0.61	0.75
3.9	0.94	0.18	0.59	0.73
4	1.00	0.14	0.59	1.00
**Left**				
2.5	0.20	1.00	1.00	0.58
2.6	0.22	1.00	1.00	0.59
2.7	0.28	0.98	0.93	0.61
2.8	0.41	0.94	0.86	0.64
2.9	0.52	0.88	0.80	0.68
3	0.57	0.81	0.72	0.68
3.1	0.59	0.77	0.69	0.68
3.2	0.70	0.73	0.70	0.73
3.3 **	0.72	0.67	0.66	0.73
3.4	0.74	0.58	0.61	0.71
3.5	0.78	0.52	0.58	0.73
3.6	0.83	0.46	0.58	0.73
3.7	0.87	0.35	0.53	0.75
3.8	0.89	0.29	0.53	0.75
3.9	0.91	0.29	0.53	0.79
4	0.93	0.15	0.49	0.67

NPV: negative predicted value; PPV: positive predicted value. ** indicates the selected optimal cutoff value.

## Data Availability

The data presented in this study are available on request from the corresponding author due to privacy reasons.

## References

[1] Aaslid R., Markwalder T.M., Nornes H. (1982). Noninvasive transcranial Doppler ultrasound recording of flow velocity in basal cerebral arteries. J. Neurosurg..

[2] Bogdahn U., Becker G., Winkler J., Greiner K., Perez J., Meurers B. (1990). Transcranial color-coded real-time sonography in adults. Stroke.

[3] Hakim M., Kawnayn G., Hassan M.S., Uddin M.N., Hasan M., Huq M.R. (2024). Transcranial Doppler in the Detection of Cerebral Vasospasm After Subarachnoid Hemorrhage. Cureus.

[4] Neulen A., Greke C., Prokesch E., König J., Wertheimer D., Giese A. (2013). Image guidance to improve reliability and data integrity of transcranial Doppler sonography. Clin. Neurol. Neurosurg..

[5] Neulen A., Prokesch E., Stein M., König J., Giese A. (2016). Image-guided transcranial Doppler sonography for monitoring of vasospasm after subarachnoid hemorrhage. Clin. Neurol. Neurosurg..

[6] Connor-Schuler R., Phillips S., Kuo E., Kandiah P., Sadan O. (2024). Feasibility and Reliability of Transcranial POCUS Color-Coded Duplex Sonography Performed by Physicians of Varied Ultrasound Experience in Diagnosing Vasospasm in Aneurysmal Subarachnoid Hemorrhage. J. Ultrasound Med..

[7] Nedelmann M., Stolz E., Gerriets T., Baumgartner R.W., Malferrari G., Seidel G., Kaps M. (2009). Consensus recommendations for transcranial color-coded duplex sonography for the assessment of intracranial arteries in clinical trials on acute stroke. Stroke.

[8] Pérez E.S., Delgado-Mederos R., Rubiera M., Delgado P., Ribó M., Maisterra O., Ortega G., Álvarez-Sabin J., Molina C.A. (2009). Transcranial duplex sonography for monitoring hyperacute intracerebral hemorrhage. Stroke.

[9] Cattalani A., Grasso V.M., Vitali M., Gallesio I., Magrassi L., Barbanera A. (2017). Transcranial color-coded duplex sonography for evaluation of midline-shift after chronic-subdural hematoma evacuation (TEMASE): A prospective study. Clin. Neurol. Neurosurg..

[10] Gerriets T., Stolz E., Modrau B., Fiss I., Seidel G., Kaps M. (1999). Sonographic monitoring of midline shift in hemispheric infarctions. Neurology.

[11] Becker G., Bogdahn U., Strassburg H.M., Lindner A., Hassel W., Meixensberger J., Hofmann E. (1994). Identification of ventricular enlargement and estimation of intracranial pressure by transcranial color-coded real-time sonography. J. Neuroimaging.

[12] Rajajee V. (2024). Transcranial Ultrasound in the Neurocritical Care Unit. Neuroimaging Clin. N. Am..

[13] Baumgartner R.W., Mattle H.P., Kothbauer K., Baumgartner R.W., Arnold M., Gönner F., Staikow I., Herrmann C., Rivoir A., Müri R.M. (1994). Transcranial color-coded duplex sonography in cerebral aneurysms. Stroke.

[14] Martin P.J., Gaunt M.E., Naylor A.R., Hope D.T., Orpe V., Evans D.H. (1994). Intracranial aneurysms and arteriovenous malformations: Transcranial colour-coded sonography as a diagnostic aid. Ultrasound Med. Biol..

[15] Baumgartner R.W., Mattle H.P., Schroth G. (1999). Assessment of ≥50% and <50% intracranial stenoses by transcranial color-coded duplex sonography. Stroke.

[16] Doijiri R., Furuya N. (2025). Visualization of Moyamoya Vessels Using Transcranial Color-Coded Duplex Sonography: A Case Report. Cureus.

[17] Rajajee V., Soroushmehr R., Williamson C.A., Najarian K., Ward K., Tiba H. (2023). Transcranial Color-Coded Sonography with Angle Correction as a Screening Tool for Raised Intracranial Pressure. Crit. Care Explor..

[18] Brisson R.T., Fernandes R.C.L., Arruda J.F.L., Rocha T.C.C.d.S.M., Santos N.d.G.D., Silva L.D., de Lima M.A.S.D., de Rosso A.L.Z. (2023). Altered Cerebral Vasoreactivity on Transcranial Color-Coded Sonography Related to Akinetic-Rigid Phenotype of Parkin-son’s Disease: Interim Analysis of a Cross-Sectional Study. Brain Sci..

[19] Vinci F., Tiseo M., Colosimo D., Calandrino A., Ramenghi L.A., Biasucci D.G. (2024). Point-of-care brain ultrasound and transcranial doppler or color-coded doppler in critically ill neonates and children. Eur. J. Pediatr..

[20] Seidel G., Kaps M., Gerriets T. (1995). Potential and limitations of transcranial color-coded sonography in stroke patients. Stroke.

[21] Krejza J., Swiat M., Pawlak M.A., Oszkinis G., Weigele J., Hurst R.W., Kasner S. (2007). Suitability of temporal bone acoustic window: Conventional TCD versus transcranial color-coded duplex sonography. J. Neuroimaging.

[22] Kollár J., Schulte-Altedorneburg G., Sikula J., Fülesdi B., Ringelstein E.B., Mehta V., Csiba L., Droste D.W. (2004). Image quality of the temporal bone window examined by transcranial doppler sonography and correlation with postmortem computed tomography measurements. Cerebrovasc. Dis..

[23] Kwon J.H., Kim J.S., Kang D.W., Bae K., Kwon S.U. (2006). The thickness and texture of temporal bone in brain CT predict acoustic window failure of transcranial Doppler. J. Neuroimaging.

[24] Baumgartner R.W. (2003). Transcranial color duplex sonography in cerebrovascular disease: A systematic review. Cerebrovasc. Dis..

[25] Brisson R.T., Santos Rda S.A., Stefano L.H.S.S., Barreira C.M.A., Arruda J.F.d.L., Dias F.A., Camilo M.R., Pontes-Neto O.M. (2021). Association between Tomographic Characteristics of the Temporal Bone and Transtemporal Window Quality on Transcranial Color Doppler Ultrasound in Patients with Stroke or Transient Ischemic Attack. Ultrasound Med. Biol..

[26] Lin Y.P., Fu M.H., Tan T.Y. (2015). Factors Associated with No or Insufficient Temporal Bone Window Using Transcranial Color-coded Sonography. J. Med. Ultrasound.

[27] Gerriets T., Seidel G., Fiss I., Modrau B., Kaps M. (1999). Contrast-enhanced transcranial color-coded duplex sonography: Efficiency and validity. Neurology.

[28] Bazan R., Braga G.P., Luvizutto G.J., Hueb J.C., Hokama N.K., Bazan S.G.Z., Nunes H.R.d.C., Leite J.P., Pontes-Neto O.M. (2015). Evaluation of the Temporal Acoustic Window for Transcranial Doppler in a Multi-Ethnic Population in Brazil. Ultrasound Med. Biol..

